# Supplementary material for: Evaluating an internet-delivered fear conditioning and extinction protocol using response times and affective ratings
Source: Sci Rep. 2022 Mar 7;12:4014. doi: 10.1038/s41598-022-07999-3 (PMC8901755; doi:10.1038/s41598-022-07999-3)
Supplement: Supplementary file 1 — Supplementary Information. [file 41598_2022_7999_MOESM1_ESM.pdf]

Supplementary material

Evaluating an internet-delivered fear conditioning and extinction protocol using response times and affective ratings.

Authors: Johannes Björkstrand[1*], Daniel S. Pine[2], & Andreas Frick[3]

Affiliations:
[1]Department of Psychology, Lund University, Lund, Sweden
[2]National Institute of Mental Health (NIMH), Bethesda, Maryland, USA
[3]The Beijer Laboratory, Department of Neuroscience, Psychiatry, Uppsala University, Uppsala, Sweden

*Corresponding author: johannes.bjorkstrand@psy.lu.se

Table S1. Descriptive statistics for missed responses. Values show the average proportion of missed trials separated by phase and stimulus type.

	N	Mean (%)	SD (%)
Acquisition: CS-	70	2.5	5.2
Acquisition: CS+	70	1.5	4.1
Extinction: CS-	63	1.6	3.6
Extinction: CS+	63	1.3	4.8
Reinstatement: CS-	61	1.4	4.6
Reinstatement: CS+	61	1.4	5.2
Total: CS-	61	1.9	3.1
Total: CS+	61	1.3	3.4
Total: Acquisition	70	2.0	3.5
Total: Extinction	63	1.5	3.3
Total: Reinstatement	61	1.4	4.3

Table S2. Percentage of missed responses were similar across phases and stimulus types. Differences in percentage of missed response across stimulus category and experimental phase were analyzed with the Wilcoxon signed rank test. Test values are presented in the table.

	N	W	p
CS+ vs CS-: Acquistion	70	68	.151
CS+ vs CS-: Extinction	63	45.5	.621
CS+ vs CS-: Reinstatement	61	18	1
Acquistion vs Extinction	63	167	.186
Acqusition vs Reinstatement	61	159	.296
Extinction vs Reinstatement	61	72.5	.834

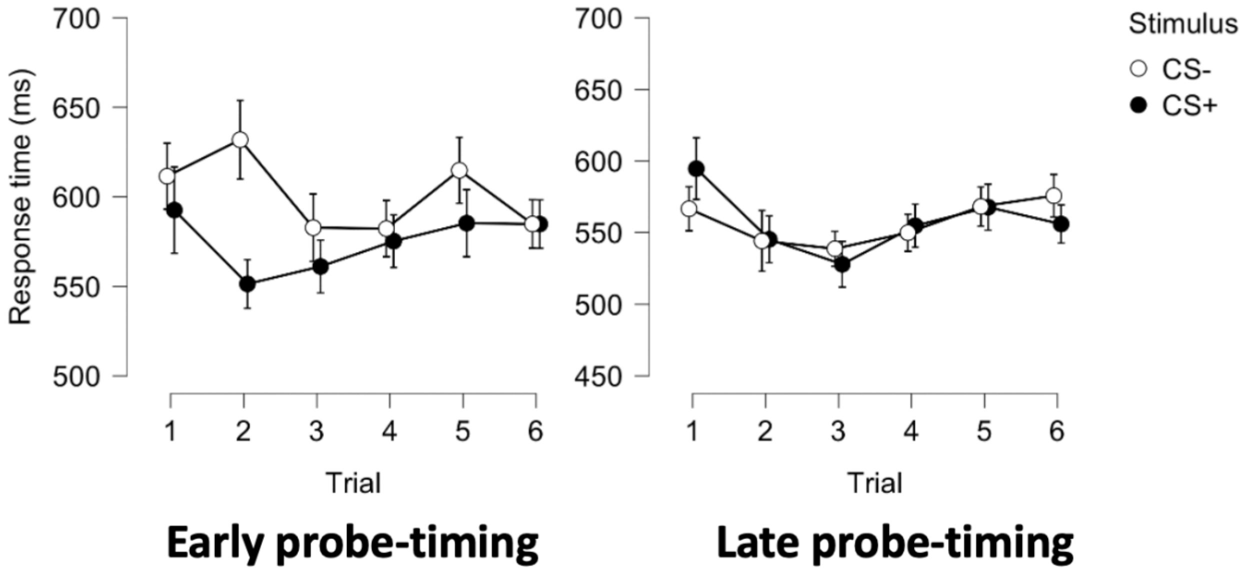

Figure S1. Trial by trial response times to early and late auditory probes during presentation of conditioned stimuli (CS) in the acquisition phase. *Early probes were presented 500ms and late probes 2500ms after CS onset. Points and error-bars indicate means and SEMs.*

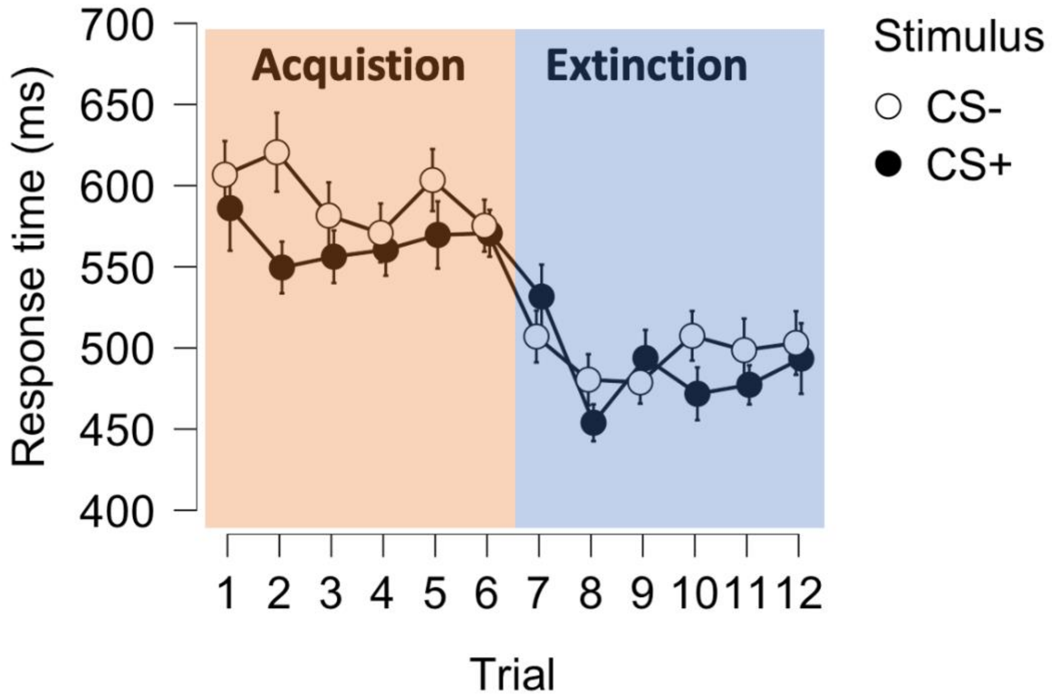

Figure S2. Trial by trial response times to early auditory probes during presentation of conditioned stimuli (CS) in the acquisition and extinction phase. *Points and error-bars indicate means and SEMs.*
